# Antiviral, Antimicrobial and Antibiofilm Activity of Selenoesters and Selenoanhydrides

**DOI:** 10.3390/molecules24234264

**Published:** 2019-11-22

**Authors:** Gabriella Spengler, Annamária Kincses, Tímea Mosolygó, Małgorzata Anna Marć, Márta Nové, Márió Gajdács, Carmen Sanmartín, Helen E. McNeil, Jessica M.A. Blair, Enrique Domínguez-Álvarez

**Affiliations:** 1 Department of Medical Microbiology and Immunobiology, Faculty of Medicine, University of Szeged, Dóm tér 10, 6720 Szeged, Hungary; spengler.gabriella@med.u-szeged.hu (G.S.); kincses.annamaria@med.u-szeged.hu (A.K.); mosolygo.timea@med.u-szeged.hu (T.M.); marcmalgorzata@gmail.com (M.A.M.); nove.marta@med.u-szeged.hu (M.N.); 2 Department of Pharmacodynamics and Biopharmacy, Faculty of Pharmacy, University of Szeged, Eötvös Utca 6, 6720 Szeged, Hungary; gajdacs.mario@pharm.u-szeged.hu; 3 Department of Pharmaceutical Technology and Chemistry, School of Pharmacy and Nutrition, University of Navarra, Irunlarrea 1, 31008 Pamplona, Spain; sanmartin@unav.es; 4 Instituto de Investigación Sanitaria de Navarra (IdiSNA), Irunlarrea 3, 31008 Pamplona, Spain; 5 Institute of Microbiology and Infection, College of Medical and Dental Sciences, University of Birmingham, Birmingham B15 2TT, UK; H.E.Smith@bham.ac.uk (H.E.M.);; 6 Instituto de Química Orgánica General (IQOG-CSIC), Consejo Superior de Investigaciones Científicas, Juan de la Cierva 3, 28006 Madrid, Spain

**Keywords:** selenoesters, selenoanhydrides, antivirals, biofilm inhibitors, antibacterials, antifungals, HSV-2, *Staphylococcus aureus*, *Salmonella* Typhimurium, *Candida* spp.

## Abstract

Selenoesters and the selenium isostere of phthalic anhydride are bioactive selenium compounds with a reported promising activity in cancer, both due to their cytotoxicity and capacity to reverse multidrug resistance. Herein we evaluate the antiviral, the biofilm inhibitory, the antibacterial and the antifungal activities of these compounds. The selenoanhydride and 7 out of the 10 selenoesters were especially potent antiviral agents in Vero cells infected with herpes simplex virus-2 (HSV-2). In addition, the tested selenium derivatives showed interesting antibiofilm activity against *Staphylococcus aureus* and *Salmonella enterica* serovar Typhimurium, as well as a moderate antifungal activity in resistant strains of *Candida* spp. They were inactive against anaerobes, which may indicate that the mechanism of action of these derivatives depends on the presence of oxygen. The capacity to inhibit the bacterial biofilm can be of particular interest in the treatment of nosocomial infections and in the coating of surfaces of prostheses. Finally, the potent antiviral activity observed converts these selenium derivatives into promising antiviral agents with potential medical applications.

## 1. Introduction

Oxidative stress plays an important role in viral infection and viral pathogenesis. The majority of viruses causes an increase in the levels of reactive oxygen species (ROS) and reactive nitrogen species (RNS) in the infected cells [1,2,3]. This increase can be caused by the immunological response against the infection (as the release of pro-oxidant cytokines or the recruitment and activation of phagocytic cells) [1] or by the intrinsic capacity of viral components to generate ROS [1,2,3,4,5]. As an example, RNA viruses can generate chronic oxidative stress [4], and certain viruses can even exploit many redox-regulated intracellular signaling pathways, to replicate inside the host cells [5]. Thus, compounds with antioxidant activity can prevent the viral action or at least can attenuate the symptoms [2,5].

In this context, selenium (Se) and the Se-containing compounds are known antioxidants [6,7,8]. This chalcogen is the crucial element that makes possible the antioxidant activity of the glutathione peroxidase, the enzyme that enables the deactivation of the hydrogen peroxides [7,8]. In line with this, patients with persistent viral infections such as HIV-1 (Human Immunodeficiency Virus) infection, usually show high oxidative stress levels. Thus, the reduction of this chronic oxidative stress through different antioxidants as Se can reduce the virulence of these viral infections [7,8]. In addition, the Se deficiency, combined with the infection with coxsackievirus, leads to the development of the Keshan disease, a severe congestive cardiomyopathy [8].

Thus, the broad relationship between Se deficiency and viral infections is well-known [7,8]. The antiviral properties of certain selenocompounds have also been described [9,10,11,12,13,14,15,16,17,18]. For instance, ebselen prevents the replication of the hepatitis C virus through the inhibition of a viral helicase [9], and diphenyl diselenide exerts antiviral activity against Herpes Simplex Virus-2 (HSV-2) [10]. In addition, benzoisoselenazol-3-(2*H*)-ones showed a more potent antiviral activity than their non-Se analogues against Herpes Simplex Virus-1 (HSV-1), Encephalomyocarditis Virus (ECMV) and Vesicular Stomatitis Virus (VSV) [11]; sodium selenite blocks the replication of hepatitis B virus in liver cancer cell lines [12], and biogenic Se nanoparticles (SeNPs) obtained from actinobacterial strains showed a potent antiviral activity against dengue virus [13]. Moreover, SeNPs functionalized with antiviral agents (ribavirin, amantadine and oseltamivir) reduced the effects of the infection by H1N1 influenza virus [14,15,16]. Finally, the Se-analogues or derivatives of antiviral drugs as selenoacyclovir, selenogancyclovir and selenazole-substituted ritonavir exerted a potent and moderate activity against HSV-1 [17], Human Cytomegalovirus (HCMV) [17] and HIV [18], respectively.

Among bacterial pathogens, *Staphylococcus aureus* is a globally important Gram-positive pathogen that frequently causes nosocomial and community-acquired infections. This bacterium is capable of biofilm formation; namely, it is able to attach to the surface of medical devices (pacemakers, implants, catheters) and can even colonize host tissue in case of chronic infection [19,20,21]. *Salmonella enterica* serovar Typhimurium is a human foodborne pathogen that causes acute inflammatory diarrhea, which can generate a systemic disease mostly in immunocompromised patients [22,23]. It has the ability to form biofilms on both biotic (epithelial cells) and abiotic (plastic and glass) surfaces [24]. Bacterial biofilms are a microbial community consisting of bacterial cells attached to each other and to a surface, and this community is embedded in a self-produced extracellular matrix. The ability to form biofilm is common in nosocomial bacteria. Bacteria embedded within biofilms are less sensitive to antibiotics, and this leads to increased treatment costs and may cause fatal outcomes [20]. Consequently, the discovery of new antimicrobial agents or adjuvants is a major challenge for drug development and for the fight against antibiotic resistance.

The antibacterial activity of Se-containing compounds has been described by many studies in the literature [25,26,27,28,29], and this research was mostly done by using bacteria that can cause nosocomial infections and/or could form biofilm, such as *Escherichia coli* and *S. aureus*. For example, a selenide polysaccharide showed antibacterial activity against *E. coli* [25], selenoesters (SeEst) exerted a potent action against *S. aureus* and *Chlamydia trachomatis* [26], selenazolinium salts have a potent activity against ESKAPE pathogens (*Enterococcus*, methicillin-resistant *S. aureus*, *Klebsiella pneumoniae*, *Acinetobacter baumannii*, *Pseudomonas aeruginosa* and *Enterobacter*) [27], selenodiazoles have antimycobacterial activity [28] and 2,2′-dithyenyl diselenide showed antibacterial activity against *Enterococcus faecalis* [29].

Additionally, different research groups have reported the capacity of SeNPs to inhibit or to hinder the formation of bacterial biofilm, mainly produced by *E. coli* and *S. aureus* [30,31,32,33,34,35,36]. Besides their aforementioned use as antiviral and antibacterial compounds, Se derivatives have also shown antifungal activity [29,37,38]. For example, diselenides [29], selenazoles [37] and SeNPs [38] showed activity against different fungal species.

In this context, our groups have synthesized and previously described a series of selenoanhydrides and selenoesters that have shown very promising anticancer activity [26,39,40,41,42,43], as these derivatives can act as chemopreventive agents [39], cytostatic derivatives [39], cytotoxic compounds [39,40,41,42], multidrug resistance reversers [40,41,42] and enhancers of the activity of clinical chemotherapy agents such as doxorubicin and topotecan, among others [43]. More recently, these derivatives have also proved to be antibacterial agents against *S. aureus*, *E. faecalis* and *C. trachomatis*, with the capacity to inhibit the bacterial AcrAB-TolC efflux pump [26].

**Figure 1:**
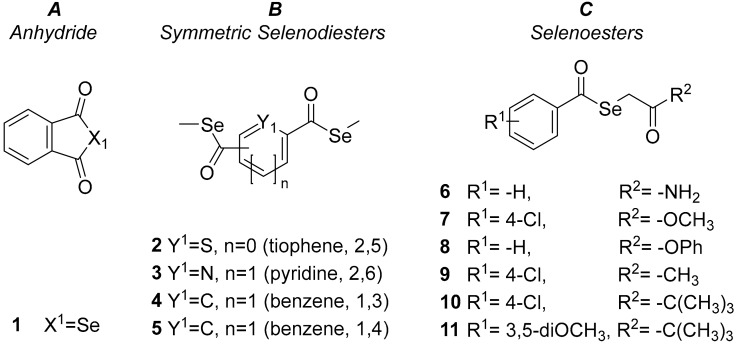
Structures of the (**A**) anhydrides, (**B**) symmetric selenodiesters and (**C**) selenoesters evaluated.

Given the promising biological activities of these compounds against cancer and specific bacterial strains, we have now explored the potential use of the selenoanhydride (SeAnh) **1** and the ten SeEst-containing compounds **2**–**11** (Figure 1) as antiviral agents against HSV-2 as antifungal agents and as inhibitors of bacterial biofilm formation.

The biofilm inhibition exerted by selenocompounds **1**–**11** was investigated by the microdilution method on Gram-positive (*S. aureus* ATCC 25923) and on Gram-negative bacteria (*S. enterica* serovar Typhimurium 14028s). The capacity of **1**–**11** to enhance the activity of antibiotics was also explored. To our knowledge, this is the first study describing the antibiofilm activity of synthetic selenocompounds, because, until now, only SeNPs have been tested against biofilms [30,31,32,33,34,35,36].

Alternatively, given these antibacterial antecedents and knowing both that selenocompounds can be correlated with their ability to act as pro-oxidants or as antioxidants [44,45] and that the SeAnh and SeEst showed chemopreventive activity in a previous study [39], we also evaluated the antibacterial activity of compounds **1**–**11** against anaerobic bacteria in oxygen-free conditions. This evaluation intended to ascertain whether the compounds exert their biological activities through the modulation of the level of ROS in their biological environments [46].

## 2. Results

### 2.1. Antiviral Activity against HSV

Before the determination of the antiviral activity of the selenocompounds, Vero cells were incubated with increasing concentrations of the compounds for 24 h. The cell viability was measured by MTT assay and IC_50_ values were evaluated (Table 1), following a procedure previously described [47]. No significant cytotoxic action was observed following the exposure of the Vero cells to concentrations of the SeAnh **1** and of SeEst **2**–**8** up to 100 μM. In contrast, compounds **9**–**11** exerted cytotoxic properties toward Vero cells; their IC_50_ value was defined at 31, 45 and 39 μM, respectively. With the purpose of avoiding the direct toxic effects of the cytotoxic compounds, concentrations of the compounds at least 6-fold lower than their IC_50_ were used in the antiviral assay.

**Figure 2:**
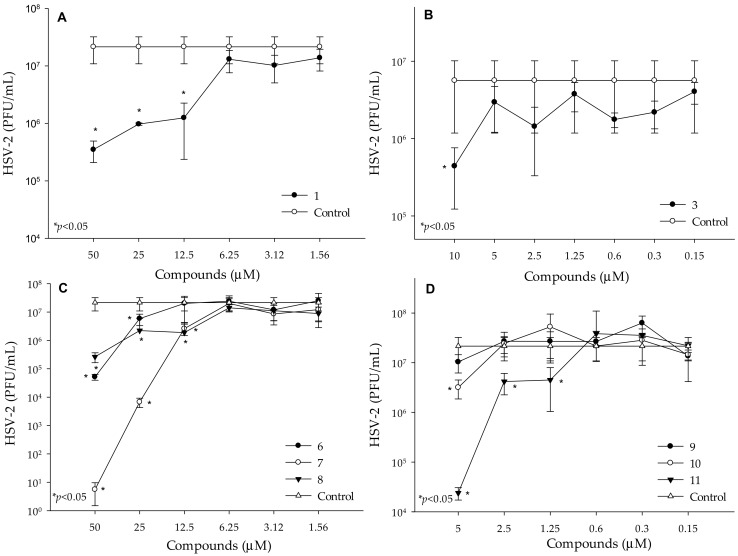
Antiviral activity of the selenocompounds (**1**, **3** and **6**–**11**) against HSV-2, drawn as follows: **A**—compound **1**, **B**—compound **3**, **C**—compounds **6**–**8**, and **D**—compounds **9**–**11.**

To determine the antiviral activity of the selenocarbonyl compounds **1**–**11**, real-time polymerase chain reaction (qPCR) was performed by using Vero cells infected with HSV-2, as previously described [48]. The SeAnh (**1**) and 6 out of the SeEst (**3** and **6**–**11**) exerted potent antiviral activity against HSV-2. The lowest concentration which exerted antiviral activity was 12.5 μM, in the case of the SeAnh (**1**) (Figure 2A). The number of HSV-2 was significantly reduced after treatment with 10 μM of selenocompound **3** (Figure 2B).

Moreover, the replication of HSV-2 was completely inhibited after treatment with 50 μM of compound **7**. The lowest concentration with antiviral activity was determined at 12.5 μM, in the case of compound **7** and **8**, while compound **6** significantly inhibited the replication of HSV-2 at 25 μM (Figure 2C). Compound **11** was the most potent anti-HSV-2 derivative, as it could inhibit the replication of HSV-2 at 1.25 μM concentration. Compound **10** also inhibited the replication of HSV-2 at the concentration of 5 μM (Figure 2D). Treatment with compounds **2**, **4** and **5**, did not reduce the number of HSV-2 (Figure S1, Supplementary Materials). The lowest concentration of the reference compound, acyclovir, which showed antiviral activity, was 10 μM (Figure S2). In addition, acyclovir could not inhibit the replication of HSV-2 completely at the examined concentrations (1.25–2500 μM). Then, among the tested selenocompounds, **10** and **11** showed antiviral activity at 8-fold and 4-fold lower concentrations than acyclovir, respectively, whereas compounds **1**, **3**, **7** and **8** exerted antiviral activities at a similar concentration to acyclovir (12.5 μM).

### 2.2. Antifungal Activity

The antifungal activity of the selenocompounds **1**–**11** against selected pathogenic yeasts was determined by using the disk-diffusion method (Table 2), measuring the respective inhibition zones as previously described [49]. The yeast strains selected for the evaluation of the antifungal activity were *Cryptococcus diffluens* (ATCC 32059), *Candida albicans* (ATCC 10231), *Candida krusei* (ATCC 14243), *Candida tropicalis* (ATCC 13803), *Candida parapsilosis* (ATCC 22019) and *Candida glabrata* (ATCC 36909).

The ketone SeEst **9**–**11** and the cyclic SeAnh **1** showed a moderate antifungal activity on the tested microorganisms in the disk-diffusion assay (Table 2) and were therefore selected for antimicrobial susceptibility testing, using the broth microdilution assay. Compounds **6** and **8** showed low antifungal activity toward *C. glabrata* and *C. krusei*, with inhibitory zones of less than 10 mm in diameter. In contrast, compounds **2**–**5** (symmetric selenodiesters) and **7** (methoxycarbonyl selenoester) did not show antifungal activity, as none of them generated an inhibition zone in any of the evaluated strains. Therefore, these compounds are not shown in Table 2, due to their lack of activity.

In the second experiment, the minimal inhibitory concentrations (MIC) of the active compounds (Those that showed an inhibition zone greater than 10 mm in the previous assay) were determined. This MIC values were calculated by using the broth microdilution method (Table 3) [50]. The MIC value of the active compounds was not determined for *C. tropicalis* or *C. glabrata* strains, because none of the four active compounds showed an inhibition zone greater than 10 mm at the 200 µM concentration in these two *Candida* fungal species.

The MIC of the four active compounds (**1** and **9**–**11**) ranged from 50 to 100 µM (Table 3). It is noteworthy that the SeAnh **1** showed the lowest MIC against *C. albicans* (50 μM), whereas *C. krusei* was more sensitive to the ketone SeEst **9** and **10** (50 μM). The lowest MIC value found in *C. diffluens* and *C. parapsilosis* was 100 μM (compounds **9** and **11**, respectively).

### 2.3. Antibacterial Activity against Anaerobes

The determination of the antibacterial activity against anaerobic bacteria had a dual purpose. The first aim of the assay was the determination of the activity of these selenocarbonyl compounds as antibacterials against different anaerobes, which are a group of pathogens where resistance is emerging steadily and for which treatment options are limited. In fact, only a few pharmacological agents (e.g., metronidazole, clindamycin and carbapenems) are able to exert antibacterial activity in anaerobic conditions [52]. The second aim was to test the activity of compounds **1**–**11** in anaerobic conditions, in order to determine if their mechanism of action depends on the presence of oxygen, because the literature suggests that the organoselenium compounds exert their biological activities through the modulation of the level of ROS in biological environments [46]. The antibacterial activity of the compounds was assessed in three anaerobic species: *Clostridium perfringens* ATCC 13124, *Bacteroides fragilis* ATCC 25285 and *Propionibacterium acnes* ATCC 11827. The standard disk-diffusion method was employed to determine the inhibition zones of the compounds [53]. A compound was considered inactive when the diameter of the inhibition zones was smaller than 10 mm in the two concentrations tested (200 and 10 mM).

The selenocompounds (**1**–**11**) did not inhibit the growth of anaerobic Gram-positive or Gram-negative bacteria at the two tested concentrations, as the diameter of the inhibition zones was always smaller than 10 mm.

### 2.4. Antibiofilm Activity against S. Aureus and S. Typhimurium

The antibacterial activity against *S.* Typhimurium exerted by the selenocompounds (**1**–**11**) was assessed. The antibacterial effect of these derivatives against *S. aureus* ATCC was determined previously [26]. Only compounds **9** (MIC: 3.12 μM) and **10** (MIC: 25 μM) had an antibacterial effect (MIC: 100 μM or >100 μM, respectively) on *S. aureus* ATCC 25923. According to the bibliography, ciprofloxacin has an MIC of 0.125 μg/mL (0.377 μM) against *S.* Typhimurium [54].

**Figure 3:**
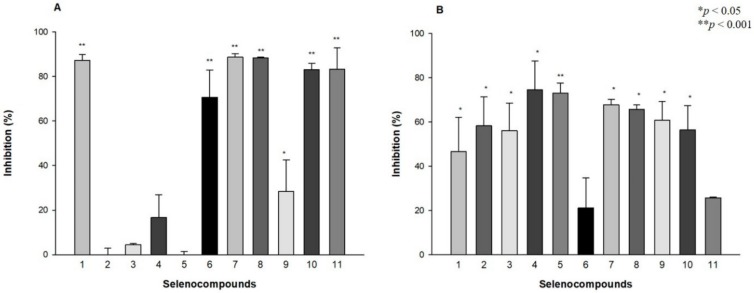
Antibiofilm effect of selenocompounds on *S. aureus* ATCC 25923 (**A**) at 50 μM (the following compounds were applied in different concentrations: **9** at 1 μM, **10** at 10 μM and **11** at 25 μM) and *S. enterica* serovar Typhimurium 14028s (**B**) at 50 μM.

The antibiofilm effect of selenocompounds on *S. aureus* and *S.* Typhimurium was evaluated. Selenocompounds **1**, **6**–**8** and **10**–**11** significantly inhibited (>70%) the biofilm formation of *S. aureus*. Compounds **1** and **6**–**8** were the most effective at 50 μM, whereas the ketone-containing SeEst **10** at 10 μM and **11** at 25 μM were effective against *S. aureus* biofilm (Figure 3A). Except for compounds **6** and **11,** all derivatives showed significant (>45% or higher) biofilm inhibition at 50 μM on *S.* Typhimurium. The most potent selenocompounds with antibiofilm effect were **4** and **5** at 50 μM, showing 75% and 73% of inhibition, respectively (Figure 3B).

### 2.5. Antibiofilm Activity against S. Aureus and S. Typhimurium in Combination with Antibiotics

The combination effect between the antibiofilm compounds and ciprofloxacin (CIP) against *S. aureus* was assessed and the combined activity with tetracycline (TET) was evaluated on *S.* Typhimurium, selecting in each separate experiment the five most active compounds (**1**, **7**, **8**, **10** and **11** for *S. aureus*; **4**, **5**, **7**, **8** and **9** for *S.* Typhimurium).

**Figure 4:**
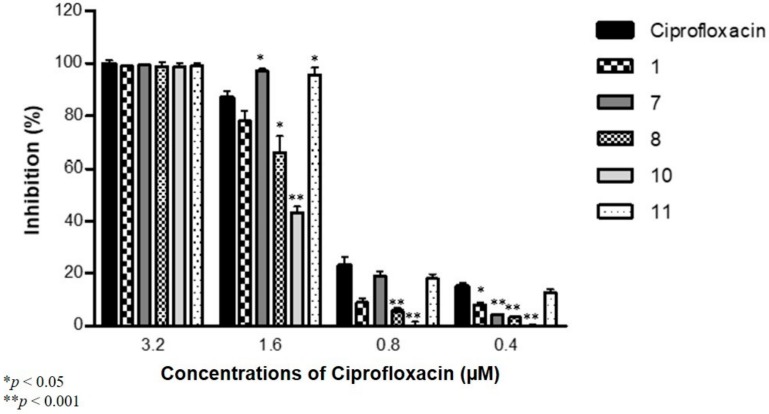
Antibiofilm effect of ciprofloxacin and selenocompounds (½ MIC; columns: compounds **1**, **7**, **8**, **10** and **11**) on *S. aureus* ATCC 25923.

Most of the compounds at a 50 µM concentration reduced the antibiofilm effect of CIP against *S. aureus* at the concentrations of 0.4, 0.8 and 1.6 μM. The exceptions were compounds **7** and **11**, which significantly increased the activity of CIP at 1.6 µM on *S. aureus* ATCC 25923 (Figure 4). No significant variations over the antibiofilm effect were exerted by CIP at a concentration of 3.2 μM.

**Figure 5:**
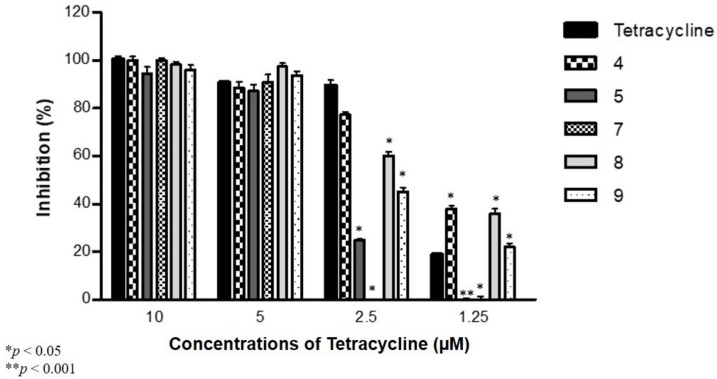
Antibiofilm effect of tetracycline and selenocompounds (½ MIC; columns: compounds **4**, **5**, **7**, **8** and **9**) on *S. enterica* serovar Typhimurium 14028s.

Compounds **4**, **8** and **9** showed significantly higher antibiofilm effects in the presence of 1.25 µM of TET; furthermore, compounds **5**, **7**, **8** and **9** in the presence of 2.5 µM of TET significantly reduced the activity of the antibiotic compared to TET alone against *S.* Typhimurium (Figure 5). No significant variations of the activity of TET were observed when TET was administered at concentrations of 5 or 10 μM.

## 3. Discussion

### 3.1. Antiviral Activity against HSV

As commented in Section 2.1, the selenocompounds evaluated herein showed a potent action against HSV-2, with SeEst **11** having the most potent antiviral activity at concentration of 1.25 µM. SeAnh (**1**) and SeEst (**3** and **6**–**10**) were also promising anti-HSV-2 compounds. All of them exerted a selective action against HSV-2: compounds **1**, **6**, **7**, **8**, **10** and **11** showed a significant antiviral activity at concentrations of 12.5, 50, 12.5, 12.5, 5 and 1.25 μM, respectively, whereas their IC_50_ values in Vero cells were at higher concentrations: 100, 100, 100, 100, 45 and 39 μM, respectively. This means that antiviral activity is observed at concentrations 8-, 2-, 8-, 8-, 9- and 31-fold lower, respectively, than the ones at which cytotoxic effects are observed in the host cells.

The high selectivity of the compounds can be explained by the fact that these derivatives generate ROS. In normal cells with an adequate ROS level, a ROS-generating drug increases the ROS levels, but without reaching the critical threshold at which cells triggers the apoptotic or necrotic processes. Nevertheless, in a cancer or an infected cell (like the case of the Vero cells infected by the HSV-2), the ROS balance is shifted toward this threshold, and the additional ROS released by the action of the ROS-generating drug can exceed the limit, causing the death of the cell, as described by Jamier et al. [55]. In line with these activity observations in antiviral assays, the SeAnh **1** and the ketone-containing SeEst **9**–**11** are pro-apoptotic compounds, as reported previously [40,41]. Interestingly, SeAnh **1** is a reactive compound that can be hydrolyzed easily [39], releasing ionic species of Se that can then exert their pro-oxidant action. The SeEst, although also capable of being hydrolyzed, are less reactive, as the hydrolysable group is protected inside the structure of the molecule, and thus these compounds are not such strong pro-oxidants like **1**.

Interestingly, some structure activity relationships can be extracted, but with the caveat that this is based on a small number of compounds. Among the SeEst, ketone derivatives **10** and **11** are potent and selective antiviral compounds that exert their antiviral action against HSV-2 at concentrations in the low micromolar range. When this ketone is replaced by a methyl oxygen ester or by an amide, the detected antiviral activity is lower, as the effect is observed if the concentrations are in the range of 12.5 to 50 μM. Finally, selenodiesters **2**–**5** did not show relevant antiviral activity in the concentrations assayed.

### 3.2. Antifungal Activity

Although only four compounds exerted a moderate antifungal activity, the results show an interesting pattern of selectivity, since some of the tested compounds were effective against *C. krusei* and *C. parapsilosis*. Both organisms have attributes that make the administration of the appropriate antifungal therapy problematic, because *C. krusei* is not susceptible to fluconazole and *C. parapsilosis* has reduced susceptibility to the echinocandins. These drugs are important agents in the treatment of systemic fungemia, and both *C. krusei* and *C. parapsilosis* are multidrug resistant opportunistic fungal pathogens [56]. In the foreseeable future, the treatment of resistant *non-albicans Candida* (NAC) will present serious issues for clinicians; therefore, the importance of research in this area should be highlighted. In spite of the modest activity found, these results are of particular interest, since optimization of this preliminary activity of the most active Se derivatives could lead to novel antifungal selenocompounds active at low micromolar concentration range.

### 3.3. Antibacterial Activity against Anaerobes

The lack of activity of the selenocompounds **1**–**11** in an anaerobic atmosphere suggests that the ROS-modulation activity of these compounds requires the presence of oxygen to trigger the cellular processes that are ultimately responsible for their antibacterial activity.

A second possible explanation of these results is that the compounds may not be able to get inside bacterial cells in anaerobic conditions, as has been described for aminoglycosides [57].

The lack of activity of these selenocarbonyl compounds against anaerobes suggests that their capacity to act as antioxidants is related to the formation or the scavenging of ROS in aerobic media [39,44,45]. This fact is interesting, as it improves the knowledge of the mechanism of action of the compounds and highlights the ambivalence of Se in the ROS modulation, frequently observed in the evaluated selenocompounds with anticancer activity. On one hand, Se is crucial to the removal of the free radicals in cellular media, through its incorporation in the form of selenocysteine to the different glutathione peroxidases. On the other hand, certain Se-containing derivatives can generate ROS and these ROS can then trigger apoptotic processes, induce mutations, or generate DNA breaks, among other effects [39,41,44,45].

### 3.4. Antibiofilm Activity against S. aureus and S. Typhimurium

Obtained results indicated that the SeAnh **1** and the SeEst containing a carbonyl group in the alkyl moiety bound to the Se atom (**6**–**8, 10** and **11**) showed a more potent antibiofilm activity against the Gram-positive *S. aureus* than the exerted by the dimethyl selenodiesters **2**–**5**, showing the relevance of this –COSeCH_2_CO– moiety for the antibiofilm activity. Furthermore, compounds **10** and **11** exerted this antibiofilm activity against *S. aureus* at a lower concentration (10 and 25 μM, respectively).

Interestingly, the dimethyl selenodiesters **4** and **5**, which contain a phenyl ring, were the most potent inhibitors of the biofilm formation in the Gram-negative *S.* Typhimurium at a 50 μM concentration, indicating the promising potential of these two difunctionalized derivatives against the assayed Gram-negative species. The derivatives **7**–**10** (with a –COSeCH_2_CO– moiety) and the dimethyl heteroarylselenodiesters **2** and **3** also exerted a biofilm inhibiting activity higher than 50% in *S.* Typhimurium. The observation that the changes in the compounds’ structure are able to modulate the antibiofilm activity is very interesting and suggests that we may be able to improve the activity further and ascertain the structure relationships behind this differential action. This fact would enable the potential discovery of more-potent antibiofilm inhibitors against *S*. Typhimurium and against *S. aureus*.

### 3.5. Antibiofilm Activity against S. aureus and S. Typhimurium in Combination with Antibiotics

The results showed that the synergistic antibiofilm effect of the compounds in combination with antibiotics was dependent on the concentration of antibacterial agents. However, the compounds interact in an antagonistic or in a neutral manner with the selected antibiotics and strains (ciprofloxacin and *S. aureus*, tetracycline and *S*. Typhimurium). Against *S. aureus*, the exceptions to this general trend were compounds **7** and **11** together with a 1.6 μM concentration of CIP. Furthermore, against *S.* Typhimurium, the exceptions were compounds **4**, **8** and **9,** in combination with a 1.25 µM concentration of TET.

The results indicate that combining these compounds with antibiotics may require a careful testing of specific combinations. As these derivatives showed an intrinsic antibiofilm activity, they could be used to coat surfaces for medical prostheses, like the different SeNPs discussed in the introduction, with potential applications in the reduction of the adherence of *E. coli* and *S. aureus* to surfaces, as well as in the inhibition of biofilm formation [30,36].

## 4. Materials and Methods

### 4.1. Chemistry

The cyclic SeAnh (**1**) and the ten SeEst investigated in this study (**2**–**11**) were synthesized by the group of Prof. Dr. Juan Antonio Palop, Prof. Dr. Carmen Sanmartín and Dr. Enrique Domínguez-Álvarez at the University of Navarra (Pamplona, Spain). Their synthesis and characterization were described in a previous work [39]. A resynthesis of the compounds was carried out, to obtain the amount necessary for performing the biological assays described in this work. The compounds purity and their structure were confirmed by using standard techniques in the characterization of organic compounds (elemental analysis, MS, IR, ^1^H-NMR and ^13^C-NMR), as previously described [39].

The chemicals used in this study during the determination of the biological activities were sodium dodecyl sulfate (SDS; Sigma, St Louis, MO, USA), 3-(4,5-dimethylthiazol-2-yl)-2,5-diphenyltetrazolium bromide (MTT; Sigma, St Louis, MO, USA) and DMSO (dimethyl sulfoxide; Sigma). Stock solutions of the tested compounds (Figure 1) were prepared in DMSO, and the working solutions were prepared by subsequent dilutions of DMSO stock solutions in water. None of the assayed solutions in biological experiments had a concentration >1% of DMSO, and all solutions were prepared and used the day of the assay.

### 4.2. Strains and Cell Lines Used

#### 4.2.1. Viral Strains

Herpes simplex virus-2 (HSV-2) was kindly provided by Dr. Ilona Mucsi (Department of Medical Microbiology and Immunobiology, University of Szeged) for its use in the antiviral assay. The titer of the HSV-2 was determined by plaque titration method and expressed in plaque-forming units (PFU)/mL.

#### 4.2.2. Cell Lines

Vero (African green monkey kidney) cells (ATCC CRL-1586) were purchased from LGC Promochem, Teddington, UK. The cells were cultured in Eagle’s Minimum Essential Medium (EMEM) supplemented with 10% heat-inactivated fetal bovine serum (Sigma-Aldrich, St Louis, MO, USA), 4 mM of L-glutamine (Sigma-Aldrich, St Louis, MO, USA), 1 mM of Na-pyruvate, a selection of non-essential amino acids (NEAA) and with a penicillin-streptomycin (Sigma-Aldrich, St Louis, MO, USA) mixture in concentrations of 100 U/L and 10 mg/L, respectively. Vero cells were used in the antiviral assay.

#### 4.2.3. Fungal Strains

*Candida albicans* ATCC 10231, *C. tropicalis* ATCC 13803, *C. krusei* ATCC 14243, *C. glabrata* ATCC 36909, *C. parapsilosis* ATCC 22109 and *Cryptococcus diffluens* ATCC 32059 were used as test organisms in the antifungal activity assays.

#### 4.2.4. Bacterial Strains

*Bacteroides fragilis* ATCC 25285 (Gram-negative; strict-anaerobe), *Clostridium perfringens* ATCC 13124 (Gram-positive; strict anaerobe) and *Propionibacterium acnes* ATCC 11827 (Gram-positive; aerotolerant anaerobe) were used in the antimicrobial susceptibility assay of anaerobic bacteria to the selenocompounds studied herein.

The Gram-negative biofilm forming *Salmonella enterica* serovar Typhimurium 14028s was used in the biofilm formation assay. The Gram-positive *Staphylococcus aureus* strain ATCC 25923 was purchased from ATCC and used in the biofilm formation assay.

### 4.3. Assay for Anti-HSV-2 Activity

The MTT assay was performed to quantify the cytotoxic activity of the tested compounds on Vero cells, as described previously [47]. A starting concentration of 100 μM of the selenocompounds was used, and optical density was used to determine the cell growth. Inhibitory concentration 50 (IC_50_) was calculated as the concentration at which the compounds were able to diminish Vero cells’ growth by 50%. With the aim of evaluating the anti-HSV-2 activity of the selenocompounds, Vero cells were seeded into 96-well plates and were infected with HSV-2 at a multiplicity of infection (MOI) of 0.01 and treated with the compounds in two-fold dilutions for 1 h at 37 °C. The starting concentrations of the compounds were chosen in accordance with their cytotoxicity on Vero cells. Control cultures were incubated with media without compounds. Acyclovir were used as reference compound, at an initial concentration of 2.5 mM. After a 24 h incubation period, the cultures were washed with phosphate buffered saline. Then, 100 μL of Milli-Q water (MQ) (Millipore, Billerica, MA, USA) was added to the cells, and the cultures in the plate were frozen. To determine the number of HSV-2, qPCR assay was performed, as previously described [48,58,59]. The qPCR mixture consisted of 5 μL of SsoFast™ EvaGreen^®^ Supermix (Bio-Rad, Hercules, CA, USA), 1–1 μL of forward and reverse primers (10 pmol/µL each), and 1 μL of template; in addition, 2 μL of MQ water was added to get a final volume of 10 μL. The virus inhibitory effect was evaluated by comparing virus titers obtained in the presence and absence of compounds.

### 4.4. Antifungal Activity against Pathogenic Yeasts

*Disk diffusion.* The antifungal activity of the selenocompounds against selected pathogenic yeasts were screened, measuring their inhibition zones [49]. Solutions of the selenocompounds were set at two concentrations: 200 µM (from a 10 mM stock solution) and 10 mM. The sterile filter paper discs (diameter (D) 6 mm) impregnated with the solutions (10 µL of solutions) were placed on Saboraud Dextrose Agar (SDA), inoculated with the respective suspensions of yeasts (inocula of 0.5 McFarland’s standard). The solvent (DMSO) served as negative control, and an additional plate was inoculated without disks, to serve as growth control. The inocula were used within 15 min of preparation, the disks were applied within 15 min of inoculating the plates, and incubation started within 15 min of application of the disks (according to EUCAST standards). The plates were then incubated for 72 h, under aerobic conditions, at 37 °C. Inhibition zone diameters generated by the tested compounds (including the diameter of the disc) were measured. All experiments were performed in triplicate.

*Broth microdilution method.* The compounds that showed activity in the disk-diffusion assay were further subjected to the determination of their minimum inhibitory concentrations on the relevant fungal strains. Determination of the MIC values was performed by using the broth microdilution method, according to EUCAST standards [50].

### 4.5. Antibacterial Activity against Anaerobic Bacteria

The antibacterial activity of the selenocompounds against the selected anaerobic bacterial strains was assessed by measuring their inhibition zones by standard disk-diffusion method [53]. Solutions of the selenocompounds were set at two concentrations: 200 µM (from a 10 mM stock solution) and 10 mM. The sterile filter paper discs (D = 6 mm) impregnated with the solutions (10 µl of solutions) were placed on Schaedler agar plates (SCS; bioMérieux), inoculated with the respective bacterial suspensions (inocula of 0.5 McFarland’s standard). The solvent (DMSO) served as negative control, and an additional plate was inoculated without disks as bacterial growth control. The inocula were used within 15 min of preparation, the disks were applied within 15 min of inoculating the plates, and incubation started within 15 min of application of the disks (according to EUCAST standards) [53]. The plates were then incubated for 72 h, under anaerobic conditions, in an anaerobic chamber (Baker Ruskinn anaerobic workstation). Inhibition-zone diameters generated by the tested compounds (including the diameter of the disc) were measured. All experiments were performed in triplicate.

### 4.6. Measuring Biofilm Formation Using Crystal Violet

The biofilm-forming ability of *S.* Typhimurium and *S. aureus* ATCC strains was studied in 96-well microtiter plates, using Luria-Bertani (LB) broth without salt (*S.* Typhimurium) or tryptic soy broth TSB (*S. aureus*) in the presence of selenocompounds. Initially, overnight cultures were diluted to an optical density (OD) of 0.1 at 600 nm and then added to each well, with the exception of the medium control wells and compounds were added individually at ½ MIC. The final volume was 200 μL in each well. Plates were incubated at 30 °C, for 48 h, with gentle agitation (100 rpm). After the incubation period, the medium was discarded, and the plate was washed with tap water, to remove unattached cells. Then, 200 μL of crystal violet (CV; 0.1% [*v*/*v*]) was added to the wells and incubated for 15 min at room temperature. CV was removed from the wells, and the plate was washed again with tap water. Then, 200 μL of 70% ethanol was added to each well, and the biofilm formation was determined by measuring the OD at 600 nm, using a FLUOstar Optima plate reader (BMG Labtech, Aylesbury, UK). The antibiofilm effect of selenocompounds was expressed in the percentage (%) decrease in biofilm formation. The assay was repeated a minimum of three times. The results were analyzed by using the *t*-test and *p*-values of <0.05 were considered significant.

### 4.7. Enhancement of the Activity of Antibiotics against Biofilm Formation

The chemosensitization exerted by the tested selenocompounds against biofilm formation was evaluated with antibiotics (TET or CIP) in the presence of non-inhibitory concentrations of the compounds (½ MIC) in both *S.* Typhimurium and *S. aureus* strains. The effect was determined by two-fold broth microdilution method in 96-well plates, using serial dilutions of TET and CIP. The first four rows contained two-fold dilutions of antibiotics, and combinations of the antibiotics and tested compounds were added into the last four rows. The overnight bacterial cultures were diluted to an OD of 0.1 at 600 nm and then added to each well, in 50 μL LB, without salt or TSB, with the exception of the medium control wells. The plates were then incubated at 30 °C, for 48 h, with gentle agitation (100 rpm), and the biofilm formation was determined by measuring the OD at 600 nm, using a FLUOstar Optima plate reader. The antibiofilm effect of antibiotic alone and in combination with selenocompounds was expressed in the percentage (%) of decrease in biofilm formation. The results were analyzed by using the *t*-test and *p*-values of <0.05 were considered significant.

### 4.8. Statistical Analysis

All values are given as the mean ± standard deviation determined for three replicates from three independent experiments. The statistical analysis of the data was performed with SigmaPlot for Windows Version 12.0 software (Systat Software Inc, San Jose, CA, USA), using the two-tailed *t*-test for independent samples. Differences were considered statistically significant when *p* < 0.05.

## 5. Conclusions

To conclude, the antiviral, antifungal, antibiofilm and antibacterial (against anaerobes) activities of a selenoanhydride and of selected selenoesters were evaluated, taking into account that these derivatives have shown promising anticancer, chemopreventive and multidrug resistance reversing activities in previous works. Regarding the anti-HSV activity, selenoesters **3**, **6**–**11** and the selenoanhydride **1** have shown a promising antiviral activity against HSV-2. These results are very promising and after the appropriate optimization of the structure of the active compounds, future experiments could lead to the development of very potent and selective novel antiviral agents.

Alternatively, results suggested that selenocompounds could be effective adjuvants in the treatment of infections caused by biofilm-producing strains of *S. aureus* and of *S.* Typhimurium: selenocompounds **1** and **6**–**8** at 50 μM, and **10** and **11** at lower concentrations were the most potent agents on *S. aureus*, whereas compounds **7** and **11** at 50 μM significantly enhanced the antibiofilm effect of CIP. On the other hand, in *S.* Typhimurium compounds **4** and **5** significantly decreased the biofilm mass, and compounds **4**, **8** and **9** exerted synergistic effect against *S.* Typhimurium in combination with TET at 1.25 μM. This activity is noteworthy and is in line with previous work that highlights the antibacterial activity of these compounds against *S. aureus* and *C. trachomatis*. Regarding the antifungal activity, the selenoanhydride **1** and the oxoselenoesters **9**–**11** exerted a moderate antifungal activity against resistant strains of *Candida* spp., such as *C. krusei* and *C. parapsilosis*. Finally, the evaluated compounds were inactive against anaerobic bacteria, suggesting that the mechanism of action responsible for their activity requires the presence of oxygen.

Concerning the structure-activity relationships, it has been confirmed in this study that the phthalic SeAnh **1** and the oxoselenoesters **9**–**11** are appealing structures, with potent antiviral activity, noteworthy antibiofilm activity, and moderate antifungal activity, in accordance with previous investigations that determined the multidrug resistance reversing and chemopreventive, furthermore pro-apoptotic activity in cancer models in vitro.

## Figures and Tables

**Table 1:** Cytotoxic activity of selenocompounds on Vero cells.

Compounds	IC_50_ (µM)
**1**	>100
**2**	>100
**3**	>100
**4**	>100
**5**	>100
**6**	>100
**7**	>100
**8**	>100
**9**	31
**10**	45
**11**	39

**Table 2:** Screening for antifungal activity of the selenocompounds by using the disk-diffusion method.

Cpd.	Conc. on Disk	Inhibition Zone Diameters (in Millimeters) in Different Fungal Strains					
		*Cryptococcus diffluens ATCC 32059*	*Candida* *albicans ATCC 10231*	*Candida* *tropicalis ATCC 13803*	*Candida* *krusei ATCC 14243*	*Candida* *glabrata ATCC 36909*	*Candida* *parapsilosis ATCC 22019*
**1**	200 µM	-	**14**	**3**	**11**	**2**	-
	10 mM	-	>**30**	>**30**	>**30**	**13**	**6**
**6**	200 µM	-	-	-	-	-	-
	10 mM	-	-	-	-	**6**	-
**8**	200 µM	-	-	-	-	-	-
	10 mM	-	-	-	**4**	-	-
**9**	200 µM	**12**	-	**2**	**19**	**6**	**4**
	10 mM	**29**	>**30**	**25**	>**30**	>**30**	>**30**
**10**	200 µM	-	-	-	**14**	-	**2**
	10 mM	-	>**30**	**16**	>**30**	**25**	>**30**
**11**	200 µM	**10**	-	-	-	-	**14**
	10 mM	**18**	**19**	-	**12**	**18**	>**30**
**DMSO**	2 *v*/*v*%	-	-	-	-	-	-

Cpd.: Compound. Conc.: Concentration. Values in bold denote inhibition-zone diameters > 10 mm.

**Table 3:** Minimal inhibitory concentrations (in µM) of selected Se-containing compounds and reference caspofungin (CSP) on susceptible fungal strains, using the broth microdilution method. Dates of reference are provided as a range and are calculated from the data in bibliography [51].

*Cryptococcus diffluens* *ATCC 32059*		*Candida albicans* *ATCC 10231*		*Candida krusei* *ATCC 14243*		*Candida parapsilosis ATCC 22019*	
**1**	>200	**1**	50	**1**	100	**1**	>200
**9**	100	**9**	>200	**9**	50	**9**	>200
**10**	>200	**10**	>200	**10**	50	**10**	>200
**11**	100	**11**	>200	**11**	>200	**11**	100
**CSP**	ND	CSP [51]	≤0.007–0.110	**CSP** [51]	0.055–0.229	**CSP** [51]	0.110–1.83

## Supplementary Materials

The following are available online at https://www.mdpi.com/1420-3049/24/23/4264/s1, Figure S1: Antiviral activity of the selenocompounds (2, 4, 5) against HSV-2, Figure S2: Antiviral activity of the reference compound (Acyclovir) against HSV-2.
