# Supplementary material for: Antiviral, Antimicrobial and Antibiofilm Activity of Selenoesters and Selenoanhydrides
Source: Molecules. 2019 Nov 22;24(23):4264. doi: 10.3390/molecules24234264 (PMC6930503; doi:10.3390/molecules24234264)
Supplement: Supplementary file 1 [file molecules-24-04264-s001.pdf]

Antiviral, Antimicrobial and Antibiofilm Activity of Selenoesters and Selenoanhydrides

Gabriella Spengler [1], Annamária Kincses [1], Tímea Mosolygó [1], Małgorzata Anna Marć [1], Márta Nové [1], Márió Gajdács [2], Carmen Sanmartín [3,4], Helen E. McNeil [5], Jessica M. A. Blair [5] and Enrique Domínguez-Álvarez [6,*]

SUPPLEMENTARY INFORMATION:

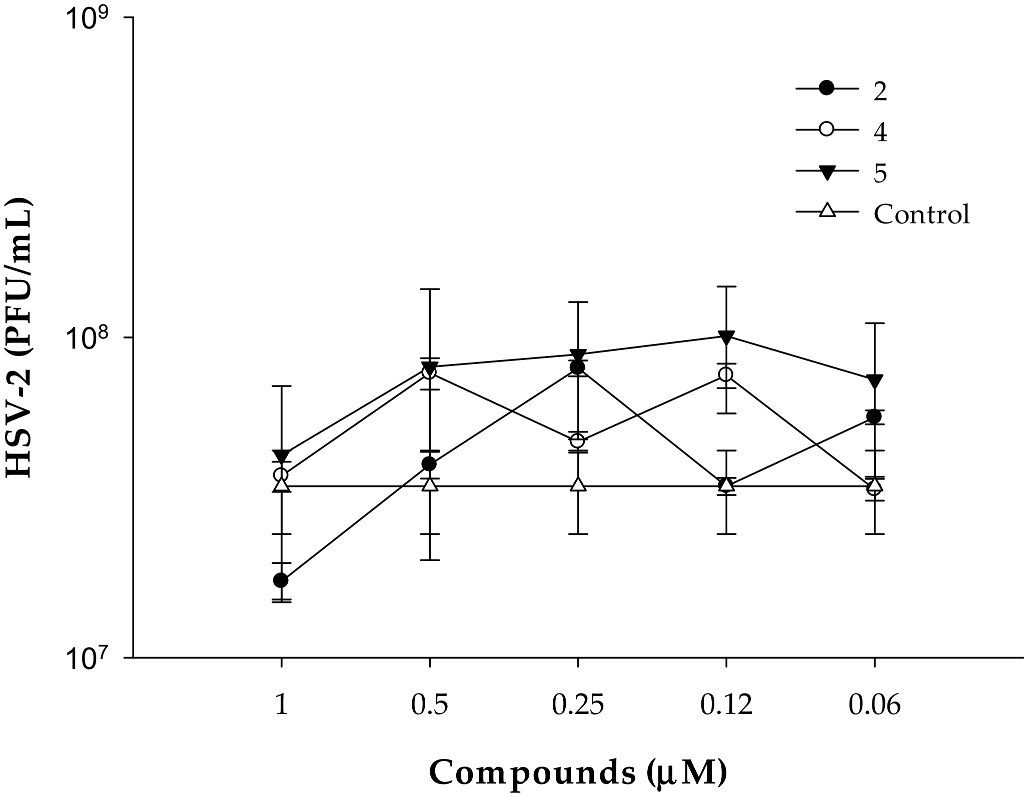

Figure S1. Antiviral activity of the selenocompounds (2, 4, 5) against HSV-2.

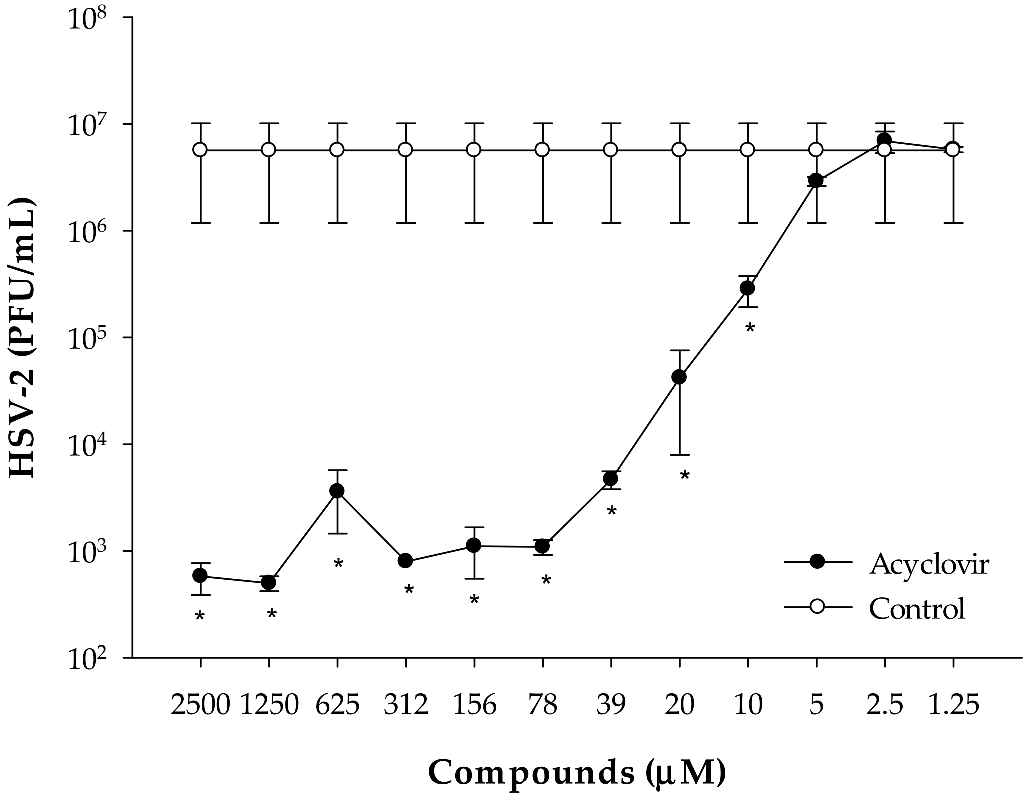

Figure S2. Antiviral activity of the reference compound (Acyclovir) against HSV-2. *p<0.05
